# The Prevention of Surgical Site Infection in Elective Colon Surgery

**DOI:** 10.1155/2013/896297

**Published:** 2013-12-19

**Authors:** Donald E. Fry

**Affiliations:** Michael Pine and Associates, 1 East Wacker Drive, No. 1210, Chicago, IL 60601, USA

## Abstract

Infections at the surgical site continue to occur in as many as 20% of elective colon resection cases. Methods to reduce these infections are inconsistently applied. Surgical site infection (SSI) is the result of multiple interactive variables including the inoculum of bacteria that contaminate the site, the virulence of the contaminating microbes, and the local environment at the surgical site. These variables that promote infection are potentially offset by the effectiveness of the host defense. Reduction in the inoculum of bacteria is achieved by appropriate surgical site preparation, systemic preventive antibiotics, and use of mechanical bowel preparation in conjunction with the oral antibiotic bowel preparation. Intraoperative reduction of hematoma, necrotic tissue, foreign bodies, and tissue dead space will reduce infections. Enhancement of the host may be achieved by perioperative supplemental oxygenation, maintenance of normothermia, and glycemic control. These methods require additional research to identify optimum application. Uniform application of currently understood methods and continued research into new methods to reduce microbial contamination and enhancement of host responsiveness can lead to better outcomes.

## 1. Introduction

Elective colon surgery continues to have the highest rate of infection at the surgical site among all elective surgical procedures. These infections span a continuum of mild superficial infection to those that are deep-seated within the abdominal cavity and pose a serious threat to the patient's survival. These infections are associated with considerable patient morbidity as a general rule and frequently require reoperation, prolonged hospitalization, and readmission to the hospital during the course of management. Surgical site infection (SSIs) have proven to be very costly in addition to the attendant patient morbidity [1, 2].

Because of the frequency and severity of these infections, there has been nearly a full century of efforts to define improved processes for prevention. Numerous methods have been proposed and employed before and during elective colon surgery to prevent infection at the surgical site. Many have strong scientific foundation, while others are driven solely by expert opinion. This presentation will attempt to provide a comprehensive context of the pathogenesis of SSI following elective colon surgery and to define those methods that have evidence to support use for prevention.

In past decades it has been the practice to refer to wound infections as separate entities from infections that occur in the abdominal cavity following colon resections. In recent years, it has become preferable to refer to all infections at the surgical site as SSIs. The infections are divided into three categories of superficial, deep, and organ/space infections (Table 1) [3, 4]. Superficial infections involve the skin and subcutaneous tissues, deep infections involve the muscle and investing fascia, and organ/space infections are in the abdominal cavity following elective colon surgery. These categorical distinctions will be important as the following discussion addresses pathogenesis and prevention.

## 2. Pathogenesis of SSI in Colon Surgery

SSIs occur as the summation of four different clinical variables in patients undergoing colon surgery. First, the *inoculum* of bacteria that contaminates the surgical site is the most important variable. It is well established from research efforts many decades ago that the greater the number of contaminating bacteria at the surgical site, the greater the probability of infection will be [5, 6]. The human colon is the repository of a huge number of bacteria. The rectosigmoid colon has colony counts that approach 10^12^ bacteria per gram of content with approximately 600 different species of bacteria [7]. There are gram negatives and gram positives, and aerobic and anaerobic species. It is not surprising that high rates of infection have occurred with surgical efforts to resect and reconstruct the human colon, since entry into the lumen of the colon regardless of preoperative preparation strategies are likely to contaminate the surgical site with millions of microbes. In a historical review, Poth noted that colon resection in the 1930s was associated with a >80% rate of incisional infection and a 10–12% mortality rate [8], with the high mortality and morbidity being mostly associated with infections that followed contamination of the large inoculum of bacteria from the colonic lumen. While infection rates are currently not that high, they continue to be reported at rates of 20–25% and are higher than other elective surgical interventions.

While the colonic lumen is the major source of bacterial contamination of the surgical site, colonic surgery is subject to all of the generally recognized other sources of bacterial contamination as well. Skin colonization can be a source that accounts for the smaller percentage (<20%) of infections that are from gram positive organisms. *Staphylococcus aureus* is the usual cause of SSIs that originate from skin or other operating room environmental contaminants of the surgical site. The prevalence of colonic contamination should not minimize an appreciation for the role of the skin and environmental sources of bacteria.

A second clinical variable that leads to SSI is the virulence of the organisms that contaminate the wound. Specific strains of bacteria have different virulence characteristics depending upon the exotoxins they produce or the character of the endotoxins in the cell wall. Aerobic gram negative organisms (e.g., *E. coli*) and anaerobic colonic species (e.g., Bacteroides fragilis) can have a synergistic relationship that results in enhanced virulence when the two species are simultaneously present in critical inoculum counts at the surgical site [9]. Antibiotic resistance should appropriately be viewed as a virulence characteristic of bacterial contaminants, and selected patients will have more resistant colonization within the colon lumen or at the level of the skin. Patients that have prolonged preoperative hospitalization, recent hospitalization for other reasons, recent antibiotic exposure for the treatment of other infections, or those who are residents of chronic care facilities will be colonized with more virulent organisms than the average patient undergoing colon surgery and can be expected to have higher rates of SSI. While the intrinsic virulence of the bacterial contamination is important, there is little that the clinician can do to manage this variable.

Environmental factors at the surgical site can decrease the number of bacteria necessary to cause SSIs, and accordingly increase rates of infections. Hemoglobin and hematoma in the surgical incision increase SSI by providing bacterial contaminants with a rich supply of ferric iron to enhance microbial replication [10]. Necrotic tissue from over-zealous use of the electrocautery or wound trauma from excessive traction pressure will increase rates of infection from lower quantities of bacteria. Foreign bodies in the wound provide contaminating microbes with a surface that cannot be cleansed by the host phagocytic cells and increase SSI rates. The foreign bodies of note for elective colon surgery would be the use of braided, nonabsorbable suture material [11]. Finally, dead space in the surgical incision serves as a dependent basin for the accumulation of serosanguinous fluids after wound closure. This drainage basin collects bacterial contaminants in an aqueous environment that cannot be readily managed by host responsiveness.

The fourth variable in SSI rates following elective colon surgery is the effectiveness of *host responses* in the patient. All surgical incisions are contaminated with bacteria and this is especially true in cases of resection of the human colon. But only a minority of surgical sites develops infection after 30 days. The responsiveness of the host makes the difference.

There are two components of the host response. There is the intrinsic, genetically program responsiveness that is poorly understood and not likely to be manipulated by preventive strategies. This was best illustrated by Sorensen et al. [12] who demonstrated that adopted children had an odds ratio of nearly 6.0 of dying from sepsis if one of their natural parents died of sepsis. This may or may not explain the infection outcome in patients where all appropriate methods of prevention were employed, but infection occurred nevertheless. Acquired impairment of host responsiveness is more likely to be encountered in the colon resection patient. These acquired variables may be chronic conditions like diabetes, chronic lung, chronic kidney, or chronic liver disease. Then there are acute conditions such as hypoxemia, hyperglycemia, hypothermia, hypoalbuminemia, or acute anemia that are associated with increased SSI rates. With SSI rates in elective colon surgery being higher than other surgical procedures, the presence of these predisposing acute conditions can be anticipated to have a significant impact on observed outcomes.

### 2.1. Organ/Space SSIs

An important but infrequently discussed aspect of SSI is the organ/space infections. In colon surgery, this is specifically the occurrence of intra-abdominal infection following resection and anastomosis. Like the surgical incision, the intra-abdominal cavity is exposed to a vast number of microorganisms at the time of surgical entry into the colon lumen. Microbial contaminants bind to the peritoneal surface, and in the majority of cases the organisms are eradicated by the innate host response.

Two circumstances may lead to the organ/space infection. First, the quantity of bacterial contamination may exceed the capacity of the host for clearance. Dense quantities of bacterial contamination that are potentially the consequence of spill of colonic contents during the procedure will ordinarily aggregate into the physiologic drainage basins of the abdominal cavity; specifically, the subphrenic/subhepatic space, the pelvis, or the paracolic gutters. These dependent areas also collect serous fluid and hematoma from the operation, which serve as environmentally enhancing variables for infection in the same way that infection occurs within the incision itself. Abscess is the consequence.

**Figure 1:**
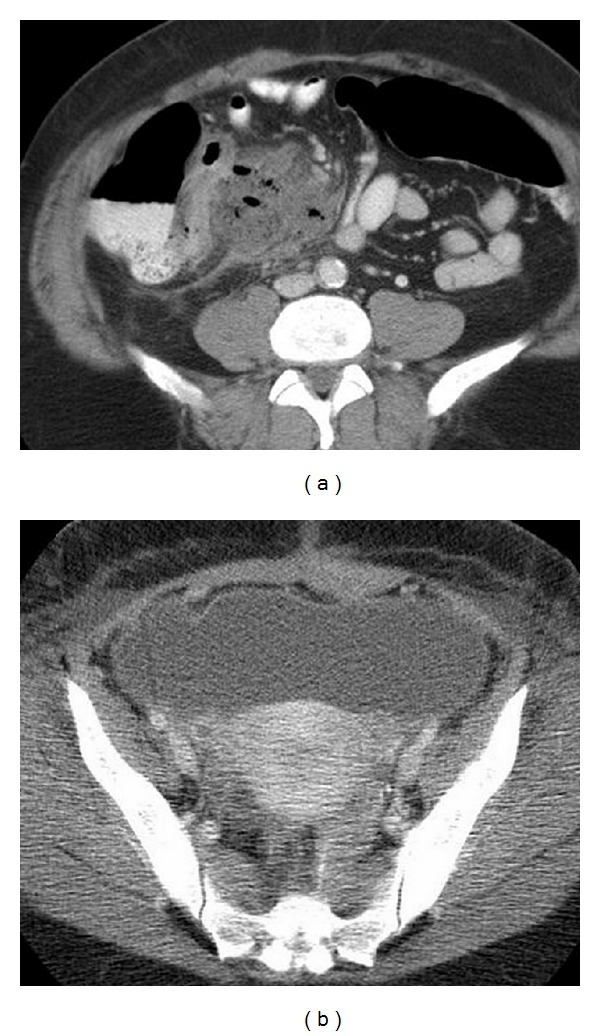
(a) Demonstrates an abdominal abscess on the right side of the abdomen following a right hemicolectomy. (b) Demonstrates a large pelvic abscess from a leaking anastomosis following a rectosigmoid colectomy.

The second circumstance for organ/space SSI is failure of the anastomosis and postoperative leakage. These leakage events are likely to occur in 3–6% of all colon anastomoses, but have higher rates when reviewing anastomosis following anterior rectal resections [13]. Defining the actual rate, like the overall rates of SSIs in any operation, has proven elusive because of variable surveillance methods for the event and the variability of definitions that are used to define when anastomotic failure has occurred [14]. Anastomotic failures with leak occur because of technical errors or excessive tension on the suture line in the construction of the anastomosis. Leaks occur because of ischemia of the tissues anastomosed together. Leaks may well occur as a consequence of infection in the anastomotic suture line because of large inocula of bacteria within the lumen or because the patient may harbor a particularly virulent composition of intraluminal microorganisms. Poor technique is commonly associated with ischemic edges of bowel in the anastomotic process and tissue ischemia likely promotes locally aggressive infection, so the reality is that all three factors in anastomotic failure may be contributors in any one case. With leakage, the patient may experience a diffuse and poorly localized fecal peritonitis that becomes a life threatening event. Leaks may lead to a localized abscess (Figures 1(a) and 1(b)). Small leaks may simply result in localized inflammation and assume very little in clinical significance. Like infections of the surgical incision, leaks occur across a wide spectrum of severity.

In summarizing the discussion on pathogenesis, it must be emphasized that there are numerous clinical variables that contribute to SSI as an outcome following colon surgery (Table 2). Many of these variables are risk factors associated with underlying comorbidities that are present in the patient that will affect host responsiveness. Other variables include the consequences of clinical interventions or behaviors that are employed in the preoperative and intraoperative care of the patient. The interaction of these numerous variables underscores why the prevention of infection in these patients requires attention to multiple clinical issues.

## 3. Microbiology of SSIs following Colon Surgery

When SSI occurs following colon surgery, it can be anticipated that the bacteriology of the infection will be those organisms that contaminated the site at the time of the operation. For most colon procedures, *E. coli* and *Bacteroides fragilis* are the most likely organisms to be encountered. Other gram negative species include *Klebsiella pneumoniae* and *Enterobacter spp*. The anaerobic species such as *B. fragilis* have the highest bacterial density in the left and retrosigmoid colon but are inconsistently cultured because they are obligate anaerobes. *Enterococcus spp.* are common in the colon but are infrequent causes of SSI in the normal host. Unusual gram negative bacteria can be anticipated if the patient has had prior antibiotic exposure or prior exposure to the healthcare environment which has resulted in alteration of their normal microflora. In these later circumstances, *Pseudomonas spp.*, *Serratia spp*., and even *Acinetobacter spp.* can be encountered. With up to 20–25% of patients in the U.S. being colonized with *Staphylococcus aureus*, these organisms will be identified in SSIs following colon surgery and many will be with the community-associated, methicillin-resistant strains [15].

Organ/space infections have similar patterns of identified pathogens. Because abscesses or diffuse peritonitis are direct consequences of intraoperative or luminal contamination, the bacteriology of these abscesses reflects the normal colonization of the human colon and makes *E. coli* and *Bacteroides fragilis* the common pathogens. One notable exception can be seen in the patient with intra-abdominal drains where *Staphylococcus aureus* can be a pathogen in abscesses due to external contamination along the drain tract.

## 4. Diagnosis and Surveillance of SSI following Colon Surgery

A key feature in an overall strategy to prevent SSI in the colon surgery patient is to have an effective surveillance program. A consistent and vigilant surveillance effort will permit the surgeon and the institution to monitor the overall rates of infection. With surveillance an objective decision can be made about improvements in outcomes, but also when clusters of infections are occurring and indicate that infection control practices need to be reviewed and modified. As is illustrated in Table 1, the definitions for SSI can be quite detailed and are often subject to interpretation by the observer.

The most common single diagnostic sign of SSI is the discharge of pus from the surgical incision. Many will use this as the only true measure of an SSI. Others will use erythema and induration of the wound, but these criteria suffer from erythema commonly being associated with an inflammatory response to stapled wound closures, and induration may or may not be detected at all in patients with a thick fat layer in the abdominal wall. Wounds may have the discharge of serous drainage which may or may not be proven culture positive for a light growth of an unlikely organism such as *Staphylococcus epidermidis*. This later pathogen may actually be a skin contaminant and not be an infectious agent, so one is left with clinical judgment to declare such a wound as being an SSI. Within a hospital, it is important to define a consistent method for defining an SSI. The greatest utility in SSI surveillance is for the purpose of improving care within the institution. Consistent monitoring of colon operations is a particularly beneficial activity because these rates will exceed all other elective procedures, and improved processes of care best lead to improved outcomes in this population of patients.

**Figure 2:**
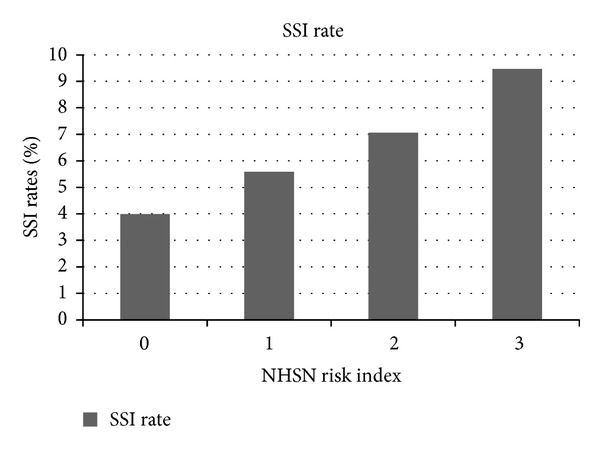
This illustrates the reported rates of SSI following colectomy for each of the NHSN index risk scores.

Rates of SSI in colon surgery, or any other operative procedure, cannot be compared across different institutions. Different definitions are used in different hospitals. The intensity of surveillance during the hospitalization will mean that those hospitals with the greatest diligence will have the highest apparent rates. Most importantly, most SSIs following colon surgery are not identified until after the patient has been discharged. Hospital based surveillance programs will not capture the postdischarge event and require special efforts to capture infectious complications after the patient leaves the hospital [16]. The differences in reported rates can be identified by my study of SSIs in an administrative dataset which represented a 20% sample of an entire year of elective surgical cases in the United States. This demonstrated an SSI rate of 3.9% [17]. The National Healthcare Safety Network (NHSN) in the U.S. observed SSI rates in elective colon surgery as between 4 and 10% (Figure 2) [18]. Three studies with prospective, 30-day surveillance by very reputable investigators have identified overall SSI rates in elective colon surgery >20% [19–21]. It is clear that different definitions and different surveillance strategies are being used.

Furthermore, different patient populations represent different risk profiles in different institutions and apparent differences may not be an equitable comparison. A somewhat simplified risk adjustment method is to use the NHSN system which segregates patients into four risk tiers depending upon the degree of intraoperative contamination, the American Society of Anesthesiologist severity score (Table 3), and the duration of the operative procedure compared to a national standard [22]. NHSN has recently developed a logistic regression model that is a far more sophisticated method to predict SSI rates [23]. Specific instructions have been issued to assist in using this model to develop a Standardized Infection Ratio (SIR) which in theory compares the local hospital infection rate to the national standard [24]. The variability in reported rates of SSI still leaves uncertainty about the accuracy of these attempts to compare infection rates among multiple hospitals. As is identified in Table 2, the number of variables to be accounted for in the identification of SSI in the elective colon operation is very large and makes the development of prediction equations a formidable task.

Public reporting of SSI rates for colon operations and other procedures has become a common goal of government and selected healthcare quality organizations. Guidelines for public reporting have even been issued [25]. Public reporting is meant to inform the public about the quality efforts of hospitals and perhaps to have this practice stimulate clinicians to a higher level of performance. Surgeons recognize all of the shortcomings of SSI surveillance and interpretation for any operation, which has led to the professional attitude that public reporting strategies are likely to disseminate misinformation more than meaningful data for use by patients.

## 5. Prevention of SSIs in Elective Colon Surgery

The prevention of SSIs in colon surgery requires the implementation of a host of preoperative and intraoperative measures. Patients undergoing colon surgery have potentially many risk factors for infection, and infection can occur from any number of specific events during the procedure (Table 2). Attention to only a select few preventive strategies while ignoring others may lead to no improvement in patient outcomes [26]. Preventive methods will be subdivided into preoperative and intraoperative strategies.

### 5.1. Preincisional Measures

#### 5.1.1. Prehospitalization Cleansing of the Surgical Site

Preoperative measures for elective colonic surgery begin with preparation of the surgical site. Considerable interest has been focused upon preoperative bathing, showering, and/or scrubbing of the proposed surgical site with antiseptic soap and/or antiseptics. Despite a host of clinical studies, meta-analyses have not demonstrated a reduction of SSI rates in clean operations or in any group of aggregated operations [27]. Studies have been highly variable with respect to the specific protocols and antibacterial agents that have been used which may explain the failure of benefit from a technique that should be of value. One recent study points to the need for repeated rather than only a single shower or scrub of the area with the antiseptic to achieve an appropriate benefit to prevention of SSIs [28]. Because the major source of microbial contamination in elective colon surgery is from the colon lumen and not the skin, it is unlikely that aggressive pre-hospitalization cleansing of the proposed surgical site will have a major impact on SSI rates.

#### 5.1.2. Prolonged Preoperative Hospitalization

The classic studies of Cruse and Foord [29] and the more recent studies by Vogel et al. [30] have demonstrated that prolonged preoperative hospitalization of 3-4 days before an operation will increase SSI rates and the incidence of other hospital-acquired infections. Prolonged preoperative hospitalization is likely to be a risk factor for case complexity and resultant higher complication rates following operation. Prolonged hospitalization also represents a sustained exposure to hospital-based pathogens and adversely affects resistant skin colonization and even the colonic microflora. Hospital-acquired colonization prior to operation is likely to increase resistance to conventional preventive antibiotics and to increase SSI rates.

#### 5.1.3. Hair Removal

Follicles of hair at the surgical site have always been viewed as a risk for bacteria and SSI. However, there is no evidence to support hair removal and a subsequent reduction in SSI rates [31]. Nevertheless, for hirsute patients undergoing elective colon surgery, a case can be made that hair removal may be of logistical value during the course of the operation. The classic studies of Alexander et al. [32] demonstrated that any removal of hair the night before operation increases the risk of SSI. Straight razor removal results in cuts and scrapes, and even electric clippers the night before the operation will potentially result in abrasions of the skin surface at the site of the proposed incision. These injuries to the skin surface the evening before the operation are likely to be sites for microbial growth of skin microflora (e.g., *Staphylococcus aureus*) and increase the probability of incisional infection. These studies also identified that straight razor hair removal in the operating room before the incision was also associated with increased SSI rates. Thus, if hair removal is deemed necessary, it should only be removed in the operating room with mechanical clippers immediately before the application of the skin antiseptic preparation. Care must be exercised even with the mechanical clippers to avoid skin abrasions with hair removal.

#### 5.1.4. Operating Room Preparation of the Surgical Site

The three major antiseptic solutions that are used for the preparation of the incision site have been chlorhexidine, povidone iodine, and isopropyl alcohol. The published guidelines from the CDC have not endorsed one skin preparation over the alternatives [33]. Isopropyl alcohol has the best antibacterial effects but it is flammable and has been identified with fires in the operating room when used in conjuction with the electrocautery. Operating room fires occur over 500 times per year in the U.S. and are consistently identified with flammable antiseptics, oxygen, and flammable drapes [34]. Chlorhexidine has been associated with better antiseptic effect than povidone iodine [35] and has been more effective in the prevention of intravascular catheter infections [36]. One review and one meta-analysis has identified better SSI prevention with chlorhexidine [37, 38].

An emerging trend has been to combine chlorhexidine (2%) with isopropyl alcohol (70%) for skin preparation. The alcohol evaporation accelerates the drying of the chlorhexidine at the time of application. A randomized trial of chlorhexidine-isopropyl alcohol compared to povidone iodine alone demonstrated a significantly lower SSI rate (16.1% versus 9.5%, *P* = 0.004) in favor of the chlorhexidine-isopropyl alcohol preparation in clean-contaminated abdominal surgery [39]. The study would have been a fairer comparison had isopropyl alcohol been added to the povidone iodine cohort of patients. One could argue that the risks of flammability with a 70% solution of isopropyl alcohol remain significant. In reviewing all of the information, chlorhexidine appears to be a better topical agent than povidone iodine. The role of adding isopropyl alcohol remains uncertain.

#### 5.1.5. Plastic Drapes/Wound Sealants

Plastic adherent drapes that are applied to the skin at the site of the incision have been used for a period of time to prevent residual skin colonization following antiseptic preparation from accessing the surgical site during the procedure. Early studies have actually reported higher infection rates with these plastic adhesive drapes which were likely due to a “greenhouse” effect of perspiration and microbial proliferation beneath the plastic [40]. Recent versions of these plastic drapes have now employed antiseptic agents (e.g., povidone iodine) on the adhesive surface and better adhesion has been engineered. However, a recent meta-analysis has failed to show any reduction in SSI rates with these newer versions [41].

A variation of the plastic drape is to use a cyanoacrylate skin sealant. The proposed surgical site is cleansed, the topical antiseptic is applied, and the antiseptic is allowed to completely dry. The topical cyanoacrylate sealant is then applied and it too is allowed to dry before the incision is then made through the seal. The concept is to seal residual skin microflora not cleansed by the preoperative skin preparation so that contamination will not occur during the remainder of the procedure. There is evidence to support this practice in clean operations [42], but it remains of certain value when the principle source of the contamination arises from within the abdominal cavity.

Another variation on the theme of a plastic drape is to use a ringed wound protector which is actually inserted into the abdomen, and the plastic drapes which are connected to a synthetic ring are then brought out of the abdomen to protect the wound interface after the incision. Such a strategy would make sense for incisional protection when the contamination is arising from within the abdomen during colon surgery. A meta-analysis has identified a benefit to these wound protectors [43], but additional clinical trials are necessary.

#### 5.1.6. Preventive Antibiotics

The use of preventive antibiotics in elective colon surgery is generally viewed as the most significant method that has improved outcomes. With the introduction of antibiotics into clinical practice following World War II, the use of antibiotics to avoid infection in colon surgery was of particular interest because of the high rates of SSI that were observed in these patients. This enthusiasm for preventive antibiotics rapidly dissipated when clinical trials in colon surgery and other operative procedures failed to demonstrate any reduction in SSI rates. The two major issues that influenced the failure for SSI reduction were the timing of administration of the antibiotic and the failure to stratify the operative cases by risk. At that time the antibiotics were given following the operation, and cases with high rates of infection (e.g., colon surgery) were studied with cases that had low rates of infection (e.g., inguinal hernia repair) which led to overall infection rates aggregating to the mean regardless of the preventive strategy.

The influence of timing upon antibiotic prophylaxis in surgery was identified in experimental studies by Miles et al. [44], and in clinically relevant experimental models by Burke [45]. The key features of preventive antibiotic use in these experimental studies were that the antibiotic needed to be in the tissue at the time of bacterial contamination of the soft tissues, and that systemic antibiotic administration after contamination had a progressively attenuated benefit. Antibiotic administration more than two hours after contamination had no impact upon the natural history of the cutaneous infection.

Polk and Lopez Mayor did the first clinical study of clean-contaminated elective surgical case with 50% colon procedures that demonstrated a statistically significant reduction in SSI rates by administration of the antibiotic (cephaloridine) before the surgical incision [46]. Patients received a second and third dose at 5 and 12 hours after the initial dose, and then all antibiotics were stopped. Subsequent studies by Stone et al. in clean-contaminated operations including colon resections showed that the antibiotic had to be administered before the surgical incision to be effective in the reduction of SSI, and furthermore, the initiation of the antibiotic after wound closure had no impact on SSI rates [47, 48].

Following these pioneering clinical trials there were a large number of reported studies that further validated the benefits of preoperative administration of preventive antibiotics. Baum and associates demonstrated the compelling results of the numerous placebo-controlled trials which showed the benefit of preoperative preventive antibiotics in colon surgery, and concluded that no further placebo-controlled trials should be performed [49]. McDonald et al. [50] performed a meta-analysis of abdominal general surgery and Song and Glenny [51] did a meta-analysis of elective colon operations only with both studies demonstrating that only preoperative antibiotic was necessary and that prolonged administration of the drug following completion of the operation did not further reduce SSI rates.

An important consideration is to understand why antibiotics given after wound closure do not improve SSI rates. Bacterial contamination on the incisional interface or over the peritoneal lining cells is promptly embedded in fibrin as part of the inflammatory response to tissue injury. During the course of the operation, the contamination of the surgical site continues from the multiple potential sources and the activation of fibrin from inflammation similarly occurs. At wound closure, the subcutaneous tissues and skin are closed and the residual wound space is also promptly populated by fibrin leaving a dense protein matrix with entrapped microbes. The fibrin matrix is impervious to systemic antibiotics from the circulation. Antibiotic effect in reduction of viable microbial counts requires that the drug must be present at the time that the fibrin is produced from the serum proteins. Antibiotics administered following the precipitation of the fibrin do not make contact with the surgical site contamination. Furthermore, the normal edema process of the activated inflammatory response continues after wound closure which results in a “halo” of increased hydrostatic pressure in the tissues about the closed incision. This makes the wound interface following closure a functionally ischemic area that excludes antibiotic delivery.

Appropriate preventive antibiotic selections for elective colon surgery are detailed in Table 4. The antibiotic selection should have activity against the likely pathogens to contaminate the surgical site. This choice would be expected to cover staphylococci as the principal skin contaminant, *E. coli* as the principal enteric gram negative rod from the colon, and *Bacteroides fragilis* as the primary colonic anaerobic pathogen of concern. This coverage profile has been identified in the second generation cephalosporin antibiotics of cefoxitin or cefotetan. This is also seen with the semisynthetic penicillins plus a *β*-lactamase inhibitor. Combination antibiotics such as a first generation cephalosporin (e.g., cefazolin) with anaerobic coverage by metronidazole or clindamycin is one choice, while a fluoroquinolone plus metronidazole or clindamycin is another. The key is to target the likely pathogens that will contaminate the surgical site.

A second consideration in the use of preventive antibiotics is the biological elimination half-life of the antibiotic. Short half-life antibiotics can be cleared rapidly and no surgical site protection may be available especially in operations that are extended for more than two hours. The issue of half-life is not only an issue relative to the length of the operation, but is also a variable in preoperative antibiotic administration that may occur 2-3 hours before the incision. It is recommended that the antibiotic selection be given as close to the time of incision as is possible to extend the duration of drug effect after the procedure is initiated. For *β*-lactam antibiotics (i.e., penicillins or cephalosporins), the antibiotic may only have a presence in the subcutaneous tissues for 2 half-lifes [52]. Thus, long half-life antibiotics (cefotetan or ertapenem) are preferred because of the longer period of surgical site coverage. For operations that extend beyond the 2 half-life limit of the antibiotic, intraoperative redosing while the incision is still open is recommended. When operations are anticipated to be extended because of operative findings early in the procedure, the surgeon should formulate a plan for the timely redosing of the antibiotic during the procedure.

Another issue that is commonly discussed but for which there is a paucity of data is the dosing of the antibiotic. Traditional dosing has been to use the same dose for all patients. The general increase in the body-mass index (BMI) of patients has raised the concern that the expanded volume of distribution of the drug in larger patients may yield inadequate incisional concentrations [53]. For bariatric and other operations in patients with a BMI > 30, consideration should be given to increasing the conventional antibiotic dose. These recommendations make sense, but it is recognized that evidence for increased dosing is lacking at present in elective colon surgery.

While the prevalence of gram positive SSIs is a smaller percentage than those infections due to colonic bacteria, the emergence of the community-acquired methicillin-resistant *Staphylococcus aureus* (CA-MRSA) has raised concerns about this pathogen in all surgical procedures [54]. This has led to many advocating screening nasopharyngeal cultures, efforts at preoperative decontamination, and the liberal use of vancomycin as a preventive antibiotic. The role of this type of surveillance program has been most actively pursued in major clean operations such as coronary artery bypass grafting or total joint replacement. Uncertainty abounds about the wisdom of these surveillance programs and what to do if the patient is found to be colonized with CA-MRSA or any staphylococcal organism [55]. The only study of comparing vancomycin against cefazolin in a high prevalence site for MRSA infections did not show a lower rate of infection with vancomycin prophylaxis in cardiac surgery [56]. At present, no studies have examined the use of MRSA coverage for elective colon surgery. MRSA surveillance has not been advocated for elective colon surgery. It should be emphasized that patients who have had a recent hospitalization, those with recent courses of systemic antibiotics, nursing home patients, and hemodialysis patients will likely have healthcare associated resistant colonization, and that a broader spectrum of preventive antibiotics should be entertained for these patients.

#### 5.1.7. Colon Preparation

While the use of preoperative preventive antibiotics in elective colon surgery is generally accepted, there is considerable disagreement about the proper colon preparation or whether the colon should undergo preparation at all in this clinical setting. With the advent of improved anesthesia and blood replacement therapy in the 1930s, efforts in colon surgery advanced. Infection within the incision and within the abdominal cavity proved to be the major complications of care. The high inoculum of bacteria within the colon lumen was appreciated as the problem and this led to mechanical bowel preparation as a potential solution to postoperative infections in these patients. From the late 1930s and the early 1940s it was understood that mechanical bowel preparation did not reduce the concentration of bacteria within the colon and did not reduce SSI rates! With the introduction of sulfa compounds, the research efforts were launched to use orally-administered poorly-absorbed antibiotics to reduce the concentration of intraluminal bacteria [57–61]. It was emphasized during all of these preliminary studies that while mechanical bowel preparation itself did not reduce bacterial concentrations or prevent SSI, it was a necessary component of antimicrobial bowel preparation to deliver the nonabsorbed antibiotic to the complete length of the colon. Retention of fecal material represented a bacterial burden that would make antimicrobial effects of the oral agent completely impractical.

During the 1950s and 1960s, varying oral antibiotics and varying mechanical preparations were used following the apparent failure of the sulfa compounds that were used. The aminoglycoside antibiotics of streptomycin [62], neomycin [63], and kanamycin [64] were evaluated because of their broad activity against colonic gram negative bacteria and because they are not absorbed from the gut. The role of anaerobes in infections following colonic contamination was not fully appreciated at this time, and Poth believed that disrupting the anaerobic environment of the colonic lumen was sufficient without specific antimicrobial coverage [8]. Mechanical preparations included a broad array of cathartics and purgatives with the duration of mechanical preparation lasting for multiple days. While testimonial evidence was given to support aminoglycoside use, no clinical trials confirmed efficacy in the reduction of SSI.

In the early 1970s, a greater appreciation of the role of anaerobic bacteria in polymicrobial infections emerged [65, 66], and investigations turned to including anaerobic coverage in potential oral antibiotic regimens. Nichols et al. [67] undertook an initial small clinical study of using oral erythromycin base with neomycin and showed an apparent benefit in reduction of SSI rates in 20 randomized patients. Most importantly, they demonstrated a dramatic reduction of 10^5^ per mL in aerobic coliform bacteria and a 10^7^ per mL reduction in *Bacteroides fragilis*. They also subsequently demonstrated high concentrations of erythromycin in the colon following mechanical bowel preparation and oral administration of the drug, and also a measurable concentration of erythromycin in the blood [68].

Using a similar rationale for coverage of colonic anaerobes as well as enteric coliforms, Washington et al. [69] at the Mayo clinic reported a three-arm trial of 200 elective colon resections that were randomized to receive (1) placebo, (2) oral neomycin only, or (3) oral neomycin plus tetracycline. All operations were performed by a single surgeon. Tetracycline was chosen because of experimental evidence by Cohn and Rives [70], which demonstrated prevention of colonic anastomotic leaks with intraluminal tetracycline, and the data presented by Cohn and Longacre [71] that proposed the use of tetracycline with neomycin for preoperative intestinal preparation. The results of the Mayo Clinic study demonstrated similar results between those patients receiving only the placebo (43% SSI) versus the neomycin group (41% SSI). However, the neomycin and tetracycline group had only a 5% rate of SSI (*P* < 0.01).

In 1977, Clarke et al. [72] reported on a randomized clinical trial from the Veterans Administration in the U.S., where elective colon resection patients (*N* = 116) were randomized to receive either a placebo or the erythromycin-neomycin oral bowel preparation following the mechanical preparation. Erythromycin was chosen as opposed to alternative choices (e.g., tetracycline) because of superior activity against *Bacteroides fragilis* as the major anaerobic pathogen of concern. These results demonstrated a 35% SSI rate in the placebo group and a 9% SSI rate in the erythromycin-neomycin group (*P* < 0.05). More important than the reduction in SSIs was the reduction of anastomotic leaks from 10% in the placebo group to none in the oral antibiotic group. Additional clinical trials validated the use of oral neomycin and metronidazole [73], or kanamycin and erythromycin [74]. The operational concept was that the oral antibiotic bowel preparation needed to cover both the aerobic and anaerobic species of the colonic microflora.

These data led to a general acceptance of the oral antibiotic bowel preparation for elective colon surgery in the U.S. Because of the parallel evolution of both the antibiotic bowel preparation and systemic preoperative preventive antibiotics in the 1970s, there was a general acceptance for using both methods together. Randomized trials of patients receiving systemic antibiotics or a placebo when patients in both arms received the oral antibiotics demonstrated reduced SSIs for both methods being used together [47, 75, 76]. Conversely, if all patients received systemic antibiotics but were randomized to oral antibiotics versus a placebo, then results likewise demonstrated improved outcomes with using both methods (Table 5) [77–83]. The rationale was that oral antibiotic bowel preparation reduced the number of bacteria contaminating the surgical site, and the systemic preoperative preventive antibiotics served as a “safety net” for those potential pathogens lodging in the soft tissues. The two methods used together were superior to either method used alone. Surveys demonstrated the preference of colorectal surgeons to use both methods together when performing elective colon surgery [84, 85].

Thus, considerable evidence supported the use of mechanical bowel preparation as a method to facilitate the delivery of intraluminal concentrations of orally administered antibiotics to reduce SSIs. The mechanical preparation employed was an intense 2-3 day preparation which often was completed with a day or two of hospitalization before the elective procedure (Table 6). Single day preparation regiments have become more popular in recent years [86, 87]. The economic pressures of managed care in the U.S. resulted in the elimination of the preoperative hospitalization time for the mechanical preparation and the administration of the oral antibiotics. Mechanical preparation became a prehospitalization event, and much shorter preparation time evolved with the use or oral polyethylene glycol solutions. Drinking four liters of solution at home on the day before the operation proved to have compliance problems because of bloating and discomfort. The oral antibiotics, particularly erythromycin, were known to cause some gastrointestinal motility issues and when added to the events of the polyethylene glycol volume of intake led to considerable patient discomfort. Retained oral solution and poor intestinal cleansing led to dissatisfaction with the necessary mechanical preparation that was essential for oral antibiotic effectiveness.

In this setting came a rush of publications that condemned the mechanical bowel preparation as being unnecessary. Prospective randomized trials flourished in Europe to validate the observations of Poth from over 70 years ago; specifically, that mechanical bowel preparation alone does not reduce SSIs (Table 7) [88–96]. The obligatory meta-analyses were done to further endorse the position that mechanical bowel preparation by itself was not of value [97]. This proliferation of trials gave the appearance of some newly discovered gem of clinical wisdom, and surgeons around the world abandoned mechanical bowel preparation and with it the oral antibiotic bowel preparation. Clinical revisionists of the historical evolution of mechanical bowel preparation and the oral antibiotic bowel preparation have set surgical progress back 40 years. The latest survey indicates that colorectal surgeons have ceased using mechanical bowel preparation and with it have stopped using oral antibiotics [98]. Even professional societies have formally recommended that mechanical bowel preparation not be done for elective colon surgery [99, 100].

Prospective randomized trials that have addressed the issue of oral antibiotics combined with the mechanical bowel preparation have largely been absent from the published literature. Lewis published a well-controlled trial which compared oral neomycin and metronidazole versus a placebo in elective colon resections where patients in both arms of the study received systemic amikacin and metronidazole [101]. The results demonstrated an SSI rate of 17% in those patients receiving only systemic antibiotics, but 5% in patients receiving both systemic antibiotics and the oral antibiotic bowel preparation. A meta-analysis in this same study demonstrated significant (*P* < 0.0001) reduction in SSI with the combination of oral antibiotic bowel preparation and systemic antibiotics compared to systemic antibiotics alone. Multiple other meta-analyses have similarly demonstrated statistically significant reductions in SSI by combining both techniques [102–104]. More recent multivariate analysis of colon resection databases have demonstrated reduced SSI rates, reduced length of hospitalization, and reduced 30-day readmission rates for patients receiving the oral antibiotic bowel preparation and systemic antibiotics together compared to patients receiving only systemic antibiotics [105–107]. Thus, the weight of clinical evidence would support the position that mechanical preparation and preventive systemic antibiotics or that preventive systemic antibiotics alone without any mechanical preparation are comparable to each other, but are suboptimal strategies compared to the use of the oral antibiotic bowel preparation with mechanical preparation that is used in conjunction with preoperative systemic antibiotics.

It is important to emphasize the correct methods to be employed in using the antibiotic bowel preparation. The mechanical preparation must be complete since the bacterial burden from unevacuated stool will negate the effective antibacterial action at the mucosa of the colon. Administration of the oral antibiotics before mechanical preparation is complete will result in the antibiotic tablets/capsules passing undissolved through the colon with no benefit to the patient. Most investigators do not believe that there is benefit to the choice of the mechanical preparation, but one study identified lower SSI rates when a phosphate-based preparation was used [108]. Experimental studies have indicated that phosphate may module the virulence of gram negative bacteria [109], but hyperphosphatemia may attend the use of this mechanical preparation [110].

There are many questions to be answered in the strategy of oral antibiotic bowel preparation. What is the best mechanical agent that will evacuate the colon completely and in a timely fashion, but without excessive discomfort for the patient? Is neomycin really necessary in the oral antibiotic regimen? One study suggests that neomycin may not be of value [111]. What are the best oral antibiotic choices? There are a host of different antibiotics that are poorly or not absorbed at all in the gut that may have value as alternatives to those that are chosen. These and many other questions in the antibiotic bowel preparation of the elective colon need to be explored instead of additional studies demonstrating the lack of benefit to mechanical preparation alone.

### 5.2. Postincisional Measures

Inoperative management of the surgical site is a critically important consideration in the prevention of SSI in elective colon surgery. While the use of antimicrobials in the perioperative period has been shown to reduce SSIs, there has been almost an exclusive dependence upon antimicrobial use to the exclusion of other methods. In the words of Altemeier from 1958, “the evidence clearly indicates, however, that antibiotic therapy cannot be depended upon to prevent the development of local infection if established surgical principles or important technical details have been ignored [112].” Poor intraoperative management can negate the benefits that preventive antibiotic strategies can provide.

#### 5.2.1. Technical Measures in Prevention

The local environment of the surgical incision is an important factor in determining whether SSI is an outcome, and these local conditions are dictated by the technical methods employed by the surgeon during the operative procedure. Minimizing tissue injury both within the incision and within the abdomen is important to prevent SSI. Overly aggressive traction creates tissue injury and rough handling of the intestine leads to local inflammation, increased risk of leakage, and organ/space SSI. Prevention of blood and hematoma requires effective hemostasis. However, over aggressive use of suture material introduces foreign bodies into the wound. Braided, nonabsorbable suture material such as silk should be avoided in the surgical incision. Excessive use of the electrocautery will leave necrotic areas within the wound and within the abdominal cavity and will result in increased infection rates. Bipolar devices have been useful in achieving the objectives of hemostasis but without excessive tissue injury. The electrocautery can be used as an alternative to the surgical knife without an increase in infection rates [113], but should be used in appropriate passes and not dwell on the tissue surface with resultant areas of black eschar. The electrocautery in not recommended for dividing intestine that will be anastomosed because of the tissue necrosis and loss of tissue perfusion that attend this practice. The management of dead space in the depths of the wound is best handled with closed suction drains that exit from a separate stabwound, never through the surgical incision itself.

#### 5.2.2. Air-Handling Systems

Air-borne bacteria as a source of wound contamination have been a long-standing concern of surgeons. Lister reputedly aerosolized carbolic acid in the operating room to remove bacterial fallout. Over 50 years ago, interest emerged in the use of ultraviolet light in the operating room to eliminate microbes in the air. An extensive multicenter study was conducted which showed no benefit to ultraviolet light use [114]. Laminar-flow air-handling systems have been used, but only have testimonial evidence to support their use even in clean operations [115]. Restricting traffic in and out of the operating room reduces the generation of air currents which may well reduce air-borne bacteria from the floor [116]. Given the large number of bacteria from the colon and a lesser but significant contamination by cutaneous microbes, it is likely that air handling strategies in general will contribute little to the reduction of SSI in elective colon surgery.

#### 5.2.3. Antibacterial Suture

Over the last 10 years, antibacterial suture material has been developed for the closure of the fascia and the subcutaneous tissues of the surgical site. Absorbable braided and monofilament sutures are coated with the antiseptic triclosan. Triclosan is a commonly used antiseptic that is contained in cosmetics and other products. It has a long record of being safe for human use [117]. The suture material has been shown to reduce bacterial growth when studied in vivo, but numerous studies from different countries have presented conflicting evidence for reduction of SSIs [118–122]. There needs to be additional studies to validate the use of this suture for the reduction of SSIs in both the surgical incision and in anastomoses of elective colon surgery.

#### 5.2.4. Irrigation Strategies

Irrigation of the surgical site is part of surgical lore, and in no place is it more evident than in elective colon surgery. Saline irrigation or irrigation with various antimicrobial or antiseptic agents has been used for decades. There is experimental evidence that contamination of a wound followed by local application of antibiotics may result in reductions in infection rates; there is no convincing clinical evidence that irrigation with antibiotics or other antimicrobial agents are of value. There is certainly no data to support topical antimicrobial irrigation if appropriate systemic antibiotics have been used. As for saline irrigation, there is reason to believe that irrigation will remove clot and fibrinous debris from the peritoneal cavity and from the surgical incision.

There has been interest in recent years about the utilization of pulsed lavage in contaminated wounds. The theory is that pulsed-lavage with its “jet stream” of force will remove the fibrin peel from the peritoneal cavity, from the interface of the surgical incision, or from other contaminated or infected tissue surfaces [123]. This method has primarily been used in contaminated orthopedic fractures, but its use in other settings of contaminated wounds including elective surgical care have been proposed. Randomized trials are necessary in the setting of elective colon surgery to make any assessment. Excessive pressure used with pulsed lavage has been a source of concern that tissue injury could be the result. Protective guards on the irrigation are also necessary to avoid potential aerosolization of microbes [124].

#### 5.2.5. Intraoperative Supplemental Oxygen

Experimental studies have documented the potential benefits of supplemental oxygen in the prevention of infection of the soft tissues following bacterial contamination [125], and a considerable amount of investigation has identified positive host response benefits from increased oxygen availability [126]. Greif et al. [127] reported a prospective randomized trial of 500 patients randomized to receive supplemental 80% inspired oxygen during and immediately following elective colon resection compared to patients receiving the conventional 30% inspired oxygen. All patients received 15 mL/kg/hr infusion rates of crystalloid solutions. The results demonstrated an 11% SSI rate in the 30% oxygen group and a 5% SSI rate in the 80% oxygen group (*P* < 0.01).

Pryor et al. [128] studied 165 abdominal laparotomy patients of which over two-thirds were colon procedures and randomized the patients to receive either 80% inspired oxygen versus 35% inspired oxygen. Their results were contradictory to the study by Greif et al. in that SSI rates were 25% in the supplemental oxygen group and only 11% in the 35% oxygen group (*P* < 0.02).

Belda et al. [129] studied 300 elective colectomy patients that were randomized to receive either 80% or 30% inspired oxygen. This study identified a 24% SSI rate in those patients inspiring 30% oxygen during and immediately following the colectomy compared to 15% in the 80% supplemental oxygen group (*P* < 0.04).

There have now been additional multiple studies in multiple different areas in using supplemental oxygen [130–134]. These and other studies either have heterogeneous patient populations, or the number of cases is too small for effective analysis. Even the usual array of meta-analyses are inconsistent in recommendations about the benefits in elective colon surgery of increased concentrations of inspired oxygen [135–139]. There remains uncertainty about the conclusions in favor of supplemental oxygen and additional studies are needed to clarify the benefits and perhaps the risks of this preventive method.

#### 5.2.6. Core Body Temperature Control

Hypothermia during operative procedures has been associated with problems of hemostasis and in the experimental laboratory with impairments of phagocytic function. Kurz et al. [140] randomized 200 elective colon surgical patients to have their intraoperative core body temperature maintained at normothermia (36.6°C) versus patients who were permitted to have the core temperature decline (34.7°C). SSIs occurred in 19% of the hypothermia patients, but in only 6% of the normothermic group.

Until recently, there has been little evidence to either support or refute the merits of maintaining normothermia in elective colon surgery, but nevertheless it has been adopted as a process measure by the U.S. Surgical Care Improvement Project (SCIP). In a retrospective study of case matched patients, Lehtinen et al. challenged whether normothermia prevented SSIs in elective colorectal surgery [141]. Melton et al. [142] have recently studied 1,008 elective colon and rectal resections, where the core body temperature was continuously monitored throughout the surgical procedure. The 30-day SSI rate in this study was 17%. With multivariate analysis, core body temperature was not a significant variable in the prediction of SSI. They have concluded that SSI is not predicted by intraoperative hypothermia. Based upon this later study, it seems appropriate that additional trials are necessary to validate the role of temperature control in elective colon surgery and other operations of the abdominal cavity.

#### 5.2.7. Glucose Control

Infectious complications in surgical patients have been associated with diabetes as a patient risk factor. Better control of the patient's diabetic disease has been traditionally associated with better outcomes following surgery. This observation led to an initial effort reported by Furnary et al. [143] to control blood sugar < 200 mgs/100 mL of diabetic patients with intraoperative and postoperative insulin infusions. This program studied over 2500 diabetic patients and resulted in a reduction of sternal wound SSIs to the same frequency as nondiabetic patients. Latham et al. [144] studied blood sugar as a risk factor and established an increasing odds ratio for SSI as the blood sugar exceeded 200 mgs/100 mL. This experience in cardiac surgery has led to the clinical questions of better glycemic control and its effects on other types of surgical interventions and what should be the target blood sugar goal to optimize outcomes but not risk hypoglycemic complications.

Hyperglycemia has multiple immunosuppressive effects upon the host [145]. Thus, perioperative hyperglycemia has been associated with surgical site infections in virtually all operations including colon resections [146]. In general surgery patients, it is hyperglycemia and not diabetes that increases all postoperative infections including SSIs [147]. Using a definition of blood sugar > 180 mg/100 mL, hyperglycemic colorectal resection patients in a patient study population of over 6,000 cases had a higher inpatient mortality rate (3.1% versus 1.0%; *P* < 0.001), required reoperation more frequently (5.9% versus 4.3%; *P* < 0.001), and more total postoperative infections (14.8% versus 9.6%; *P* < 0.001) [148]. These studies have been retrospective reviews, and prospective evaluations will need to be done that include better definition of the target blood sugar concentration for patient management. It is certain that efforts to define best practices in the intraoperative and postoperative management of glucose will be greatly aided by improved real-time measurements of blood glucose.

#### 5.2.8. Delayed Primary Closure

Delayed primary closure is a strategy for prevention of SSI if active infection or severe contamination is encountered at operation. Introduced in 1940 [149], this method entails closing the abdominal fascia following a laparotomy but leaving the skin and subcutaneous tissue open for daily management. Moist gauze dressings are used in the wound to prevent desiccation and avoid eschar formation. On the third or fourth postoperative day, if the wound interface appears free of exudate, the wound is then closed. In the setting of elective colon resection, this method should only be considered in the rare event of a major spill of colonic contents in an unprepared colon or an unanticipated abscess that is encountered during the procedure.

## 6. Postoperative Prevention of SSI

There is little evidence to support any specific methods for the prevention of SSI during the postoperative period. As has been emphasized, systemic antibiotics are not recommended nor are they proven to be of any value in the prevention of SSI. The current practices of using supplemental oxygen, core body temperature control, and glycemic control are extended into the postoperative period of time for several hours, but it is unclear whether the extension is the critical time or whether the intraoperative physiologic manipulation is the key period. The timing of use of these physiologic methods may be an important variable in the uncertainty that surrounds the benefit in using them.

Dressings are used on the wound following primary closure to avoid any potential secondary contamination from the environment. By 24 hours, the wound has a fibrin seal and at that time the primarily closed wound is not in need of dressings. In the uncommon event of a stoma being on the abdominal wall, a longer period of incisional coverage may be prudent. The reality is that infection of the surgical incision is the consequence of bacterial contamination during the procedure and secondary contamination afterwards is an uncommon occurrence.

## 7. Summary

Infection at the surgical site will continue to be a major challenge for prevention in elective colon surgery. The strong movement from the traditional open laparotomy for colectomy to greater utilization of laparoscopic-assisted techniques means that the surgical incision into the abdominal wall will be shorter and it is likely that infections may be less frequent and they may be less severe in the superficial and deep SSI categories. It is likely that organ/space infection following colonic resection and anastomosis will continue to be major sources of morbidity and mortality. Surveillance methods and definitions of SSI need to be standardized so that clinicians have a clear objectively based goal to pursue in the improvement of outcomes. Thus, there needs to be a continued use of all the accepted techniques for reduction of contamination at the surgical site and a continued vigilance in the prevention of local incisional conditions that promote infection.

New methods need to be advanced to reduce SSIs in colorectal surgery. It seems unlikely that great advances in systemic antibiotics will likely evolve to further improve outcomes. The efforts at achieving optimal physiological conditions in the host by answering the questions surrounding intraoperative supplemental oxygen, normothermia, and appropriate glucose control seem desirable. It seems appropriate that methods to prepare the colon itself prior to operation have the greatest opportunity to reduce intraoperative contamination and to potentially reduce anastomotic leaks. The area of colonic preparation needs innovative efforts in the development of effective methods for prevention and less recapitulation of studies that have limited value in advancing the outcomes of care.

## Figures and Tables

**Table 1:** Definitions of superficial, deep, and organ/space SSIs as defined by the National Healthcare Safety Network. The comments are specifically applied to elective colon surgery.

Definition	Comments specific for elective colon surgery
*Superficial SSI*: infection occurs within 30 days after any NHSN operative procedure, infection involves only skin or subcutaneous tissue of the incision, and the patient has at least one of the following. (1) Purulent drainage, with or without laboratory confirmation, from the superficial incision. (2) Organisms isolated from an aseptically-obtained culture of fluid or tissue from the superficial incision. (3) Superficial incision is deliberately opened by a surgeon and is culture-positive or not cultured, and patient has at least one of the following signs or symptoms: pain or tenderness: localized swelling; redness; or heat. A culture negative finding does not meet this criterion. (4) Diagnosis of superficial incisional SSI by the surgeon or attending physician, or other designee (nurse practitioner or physician's assistant).	(1) There are two specific types of superficial incisional SSIs. (i) Superficial Incisional Primary (SIP): a superficial incisional SSI that is identified in the primary incision in a patient that has had an operation with one or more incisions (e.g., primary laparotomy site in a colectomy). (ii) Superficial Incisional Secondary (SIS): a superficial incisional SSI in a secondary incision (e.g., second incision site of a colostomy closure). (2) Do not report stitch abscess (minimal inflammation and discharge confined to the points of suture penetration). (3) Do not report a localized stab wound or drain site infection as an SSI. (4) Do not report cellulitis by itself as an SSI. (5) Incisional SSI that extends into the fascial and muscle layers is reported as a deep incisional SSI, not a superficial SS.

*Deep SSI*: infection occurs within 30 days after elective colon resection and involves deep soft tissues of the incision (e.g., fascial and muscle layers), and the patient has one of the following. (i) Purulent drainage from the deep incision (i.e., pus) (ii) A deep incision that spontaneously dehisces or is deliberately opened by a surgeon and is culture-positive or not cultured, and the patient has at least one of the following signs and symptoms: fever (>38°C); localized pain or tenderness. A culture-negative finding does not meet this criterion. (iii) An abscess or other evidence of infection involving the deep incision that is found on direct examination, during invasive procedure, or by histopathologic examination or imaging test. (iv) Diagnosis of a deep incisional SSI by a surgeon or attending physician or other designee (nurse practitioner or physician's assistant).	(i) There are two types of deep incisional SSIs. (a) Deep Incisional Primary (DIP): a deep incisional SSI that is identified in a primary incision where multiple incisions exist (e.g., midline laparotomy and colostomy closure site). (b) Deep Incisional Secondary (DIS): a deep incisional SSI that is identified in the secondary incision where multiple incisions may exist (e.g., colostomy closure site). (ii) Infections involving both superficial and deep sites should be classified as deep incisional SSIs. (iii) The attending physician is interpreted to mean. (a) Surgeon (b) Infectious disease specialist (c) Other physician on the case (d) Emergency physician (e) Physician's designee

*Organ/Space SSI*: infection occurs within 30 days after elective colon resection and infection involves any part of the body, excluding the skin incision, fascia, or muscle layers, that is opened or manipulated during the operative procedure, and the patient has at least one of the following. (i) Purulent drainage from a drain that is placed into the organ/space. (ii) Organisms isolated from an aseptically-obtained culture of fluid or tissue in the organ space. (iii) An abscess or other evidence of infection involving the organ/space that is found on direct examination, during invasive procedure, or by histopathologic examination or imaging test. (iv) Diagnosis of an organ/space SSI by a surgeon or attending physician or other designee (nurse practitioner or physician assistant).	(i) Because an organ/space SSI involves any part of the body (excluding skin incision, fascia, or muscle layers) that is manipulated during the operative procedure, criterion for infection at these body sites must be met in addition to the organ/space SSI criteria. (ii) If a patient has an infection in the organ/space being operated on and the surgical incision was closed primarily, subsequent continuation of this infection type during the remainder of the surveillance period is considered an organ/space SSI, if organ/space SSI and site-specific infection criteria are met. (iii) Occasionally an organ/space infection drains through the incision and is considered a complication of the incision. Therefore, classify it as a deep incisional SSI. (e.g., subfascial abscess).

**Table 2:** Identifies the patient risk factors and the treatment-related risk factors that influence SSI rates in patients undergoing elective colectomy.

Patient risk factors		Treatment-related risk factors	
Advanced age	Obesity	Length of operation	Hair removal strategy
Alcoholism	Drug abuse	OR traffic	Glove/barrier failure
HIV disease	Chronic liver disease	Poor antibiotic timing	Wrong antibiotic choice
Chronic renal disease	Corticosteroids	Intraoperative “spill”	Excessive electrocautery
Chronic tobacco use	Diabetes	Skin antiseptics	Adhesive drapes
Hyperglycemia	Chronic lung disease	Contaminated instruments	Contaminated irrigation solution
Hypoalbuminemia	Malignancy	Preoperative showers	Braided suture material
Nasal colonization	Preoperative nursing home	Excessive traction/wound trauma	Wound dead space
Chronic hemodialysis	Recent hospitalization	Transfusion	Drains
Presence of stoma	ASA score	Wound hematoma	Glove starch
Resistant Bacterial Colonization	Virulent colonization	Intraoperative hypothermia	OR air handling systems
Prehospitalization antibiotics	Inflammatory bowel disease	Antibacterial sutures	Wound sealants
Prior surgical site infections	Preoperative anemia	Patient controlled analgesia	Pulsed-lavage of the surgical site
Nonsteroidal anti-inflammatory agents	Recent weight loss	Mechanical bowel preparation	Oral antibiotic bowel preparation

**Table 3:** Descriptor of the six categories that currently comprise the American Society of Anesthesiology Physical Status Classification System*.

ASA score	Description of classification	Patient example
1	Normal healthy patient	A 21-year-old, well-conditioned male athlete undergoing elective groin hernia repair
2	Patient with mild systemic disease	A 46-year-old woman with mild but controlled hypertension undergoing a laparoscopic cholecystectomy
3	Patient with severe systemic disease	A 53-year-old man with insulin-dependent diabetes and coronary artery disease undergoing elective aortofemoral bypass
4	Patient with severe systemic disease that is a constant threat to life	A 62-year-old woman on chronic renal hemodialysis undergoing emergency laparotomy for perforative diverticulitis
5	Moribund patient who is not expected to survive without the operation	A 58-year-old man with morbid obesity, type 2 diabetes, and shock, undergoing extensive debridement for streptococcal necrotizing fasciitis
6	Patient declared brain-dead whose organs are being removed for donor purposes	A 35-year-old male motorcycle accident victim with brain death and normal cardiac function, for multiorgan thoracic and abdominal organ donation

*These classes are clearly subjectively determined but have been very accurate in the prediction of risk of SSI when applied by experienced anesthesiologists.

**Table 4:** These are the preventive antibiotic choices that are currently recommended by the Surgical Care Improvement Project. The advantages and disadvantages are the authors opinion.

Drug choice (dose)	Advantages	Disadvantages
Cefoxitin (1 g)	Low toxicity cephalosporin with many years of use for prophylaxis; aerobic and anaerobic coverage.	Short biological elimination half-life (45 min); concerns about gram negative resistance.
Cefotetan (1 g)	Low toxicity cephalosporin with many years of use for prophylaxis; aerobic and anaerobic coverage. Long biological elimination half-life (4 hr)	Concerns about gram negative resistance.
Ampicillin/sulbactam (1.5–3.0 g)	Extensively used penicillin with a beta-lactamase inhibitor; good anaerobic coverage.	Short biological elimination half-life (1 hr); emerging *E. coli* resistance in up to 40% of isolates.
Ertapenem (1 g)	Extended gram negative coverage (not *Pseudomonas spp.*); long biological elimination half-life (3.5 hr).	Expense.
Cefazolin (1 g) and metronidazole (500 mg)	Good bacteriological coverage of anticipated pathogens	Limited clinical data to show effectiveness in elective colon surgery
Cefuroxime (500 mg) and metronidazole (500 mg)	Good bacteriological coverage of anticipated pathogens	Limited clinical data to show effectiveness in elective colon surgery
Aminoglycoside (gentamicin or tobramycin; 1 mg/kg) and clindamycin (300–600 mg)	A good choice for patients needing extended gram negative coverage (e.g., nursing home patients)	Unpredictable aminoglycoside pharmacology.
Quinolone (ciprofloxacin; 500–750 mg, or levofloxacin; 500–750 mg) and clindamycin (300–600 mg)	Comprehensive antimicrobial coverage of anticipated pathogens.	Limited data to validate use for prophylaxis in elective colon surgery
Aztreonam (1 g) and clindamycin (300–600 mg)	Good antimicrobial coverage of anticipated pathogens.	Aztreonam has no gram positive coverage and should not be used with metronidazole
Aminoglycoside (gentamicin or tobramycin; 1 mg/kg) and metronidazole (500 mg)	A good choice for patients needing extended gram negative coverage (e.g., nursing home patients)	Unpredictable aminoglycoside pharmacology.
Quinolone (ciprofloxacin; 500–750 mg, or levofloxacin; 500–750 mg) and metronidazole (500 mg)		

**Table 5:** This is a summary of studies comparing the oral antibiotic bowel preparation plus systemic antibiotics versus systemic antibiotics alone in elective colon surgery. Only studies with a total study population of 100 or more patients are included. The oral antibiotics used are indicated.

Author, (year)	Combined antibiotics received	Oral and IV antibiotics		IV Antibiotics only		Comments
		SWI	No. of patients	SWI	No. of patients	
Kaiser et al. (1983) [77]	Neo-erythro	2 (3%)	63	7 (12.5%)	56	*P* < 0.06; *P* < 0.05 for operations > 4 hrs in duration
Lau et al. (1988) [78]	Neo-erythro	3 (5%)	65	5 (7.5%)	67	No statistical difference
Coppa and Eng (1988) [79]	Neo-erythro	9 (5%)	169	15 (11%)	141	*P* < 0.11
Reynolds et al. (1989) [80]	Neo-metro	9 (8%)	107	26 (12%)	223	No statistical difference
Khubchandani et al. (1989) [81]	Neo-erythro	5 (9%)	55	14 (30%)	47	*P* < 0.03 (*P* < 0.05 with Yates' correction)
Taylor and Lindsay (1994) [82]	Ciprofloxacin	17 (11%)	159	30 (18%)	168	*P* < 0.11; no published evidence to support ciprofloxacin.
McArdle et al. (1995) [83]	Ciprofloxacin	8 (10%)	82	20 (23%)	87	*P* < 0.05; no published evidence to support ciprofloxacin.
Lewis (2002) [101]	Neo-metro	5 (5%)	104	17 (16.5%)	103	*P* < 0.01

**Table 6:** Demonstrates the choices of mechanical bowel preparation that has been employed in those studies where the oral antibiotic bowel preparation has been demonstrated to be effective. There are many variations on these protocols.

Washington et al., 1974 [69]	Nichols et al., 1973 [67]	One day preparation
(i) Residue-free diet for 48 hours before operation. (ii) Sodium phosphate and biphosphate 16 mL twice daily for 48 hours before operation. (iii) Two tap water enemas two days before operation. (iv) Two tap water enemas each on the morning and afternoon of the day before operation. (v) 500 mg neomycin and 250 mg tetracycline taken four times daily for 48 hours before operation.	(i) Day 1: low residue diet; Bisacodyl, 1 capsule orally at 6 p.m. (ii) Day 2: continue low residue diet; Magnesium sulfate, 30 mL. 50% solution (15 Gm.) orally at 10:00 a.m., 2:00 p.m., and 6:00 p.m; Saline enemas in evening until return clear (iii) Day 3: clear liquid diet; supplemental IV fluids as needed. Magnesium sulfate, in dose above, at 10:00 a.m. and 2:00 p.m.No enemas. Neomycin (1 gm) and erythromycin base (1 gm) at 1300 hrs, 1400 hrs, and 2300 hrs. (iv) Day 4: operation scheduled at 8:00 a.m.	*Zhu et al., 2010* [87] Day before procedure: 48 gms of sodium phosphate with 2 liters or more of water given the day before the procedure; if not clear, then saline enemas until clear with all completed by 1800 hrs. Then 2 g of neomycin and 2 g of metronidazole at 1900 and 2300 hrs. OR *Condon and Ludwig, 1995* [86] Day before procedure: 4 liters of polyethylene glycol (60 gms) and salts (Colyte, Golytely) to be completed by 1200 hrs; then neomycin 1 g and erythromycin 1 g at 1300 hrs, 1400 hrs, and 2200 hrs.

**Table 7:** A summary of the prospective randomized trials of no mechanical bowel preparation versus patients receiving mechanical bowel preparation in elective colon surgery 2000–2010. Some reports include all surgical site infections (∗), whereas others include only surgical incision infections (∗∗). Only one article concludes that there is a statistically significant difference in infection rates, which is higher in mechanically cleansed patients.

Author (Year)	No mechanical preparation		With mechanical preparation		Statistical significance
	No. of patients	Infections	No. of patients	Infections	
Miettinen et al. (2000)* [88]	129	20 (**8%**)	136	13 (**10%**)	Not significant
Bucher et al. (2005)* [91]	75	6 (**8%**)	78	17 (**22%**)	*P* < 0.03 higher with mechanical preparation
Fa-Si-Oen et al. (2005)* [90]	125	13 (**10%**)	125	16 (**13%**)	Not significant
Ram et al. (2005)** [89]	165	10 (**6%**)	164	16 (**10%**)	Not significant
Zmora et al. (2006)* [92]	129	17 (**13%**)	120	15 (**12%**)	Not significant
Jung et al. (2007)* [94]	657	106 (**16%**)	686	103 (**15%**)	Not significant
Contant et al. (2007)** [93]	684	96 (**14%**)	670	90 (**13%**)	Not significant
Pena-Soria et al. (2008)* [95]	64	11 (**17%**)	65	19 (**29%**)	Not significant
Van't Sant et al. (2010)** [96]	213	36 (**17%**)	236	39 (**16%**)	Not significant
